# A novel curved wire retraction device for endoscopic submucosal dissection

**DOI:** 10.1016/j.vgie.2021.04.006

**Published:** 2021-05-26

**Authors:** Amit Bhatt, Neal A. Mehta, Seiichiro Abe, Yutaka Saito

**Affiliations:** 1 Department of Gastroenterology, Hepatology, and Nutrition, Digestive Disease and Surgery Institute, Cleveland Clinic, Cleveland, Ohio; 2 Endoscopy Division, National Cancer Center Hospital, Tokyo, Japan

**Keywords:** ESD, endoscopic submucosal dissection

## Abstract

Video 1 Traction wire presentation and demonstration of technology in 3 endoscopic submucosal dissection cases.

Traction wire presentation and demonstration of technology in 3 endoscopic submucosal dissection cases.

Endoscopic submucosal dissection (ESD) is a well-established minimally invasive endoscopic technique for the en bloc resection of early GI malignancies.^1^ Adoption of the technique in the West has been limited by its difficult learning curve, longer procedure times, and risk of adverse events. Device innovation is needed to make the procedure easier and safer to perform. A known limitation of ESD is that the procedure is performed through a single device, and there is no “surgeon’s second hand”^2^ to provide traction on the lesion. Reliable tissue retraction exposes the submucosa, allowing for easier submucosal dissection.

We developed a novel retraction device that is simple to use but can deliver continuous retraction throughout the ESD procedure and is independent of the scope.

## Description of technology

**Figure 1:**
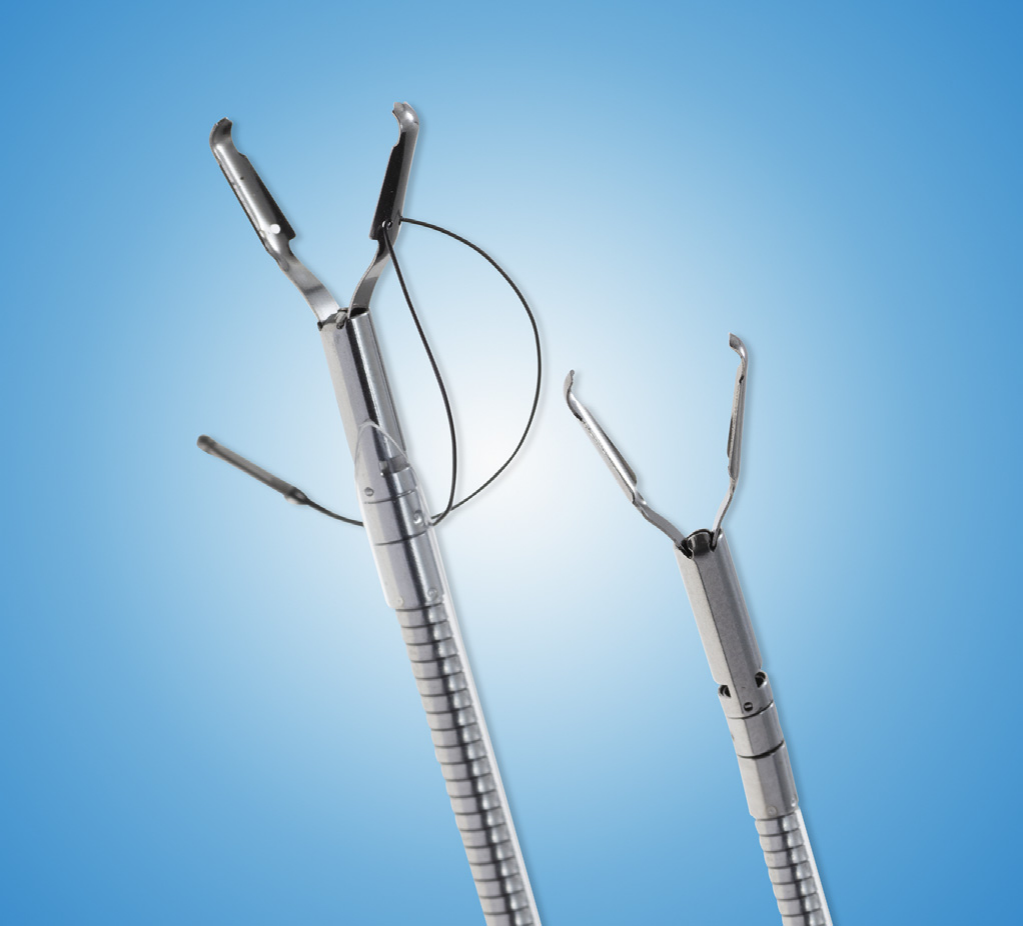
Traction wire device.

**Figure 2:**
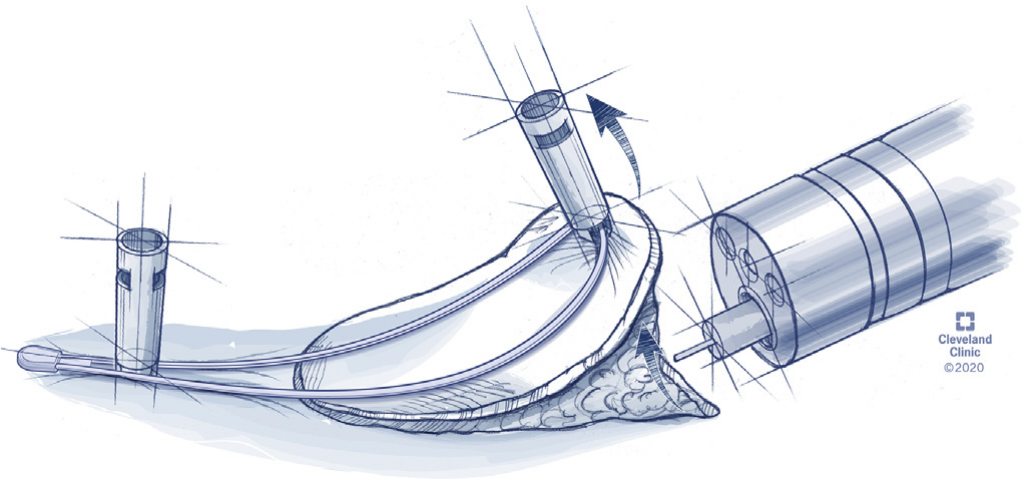
Concept drawing of traction wire device in use.

The device is commercially available as the ProdiGI Traction Wire (ERD-TW20, ERD-TW35; Medtronic, Minneapolis, Minn, USA). The device is currently commercially available in the United States and has received clearance in Europe and Japan and will soon be commercially available there. The device is a traction wire system consisting of a curved wire loop attached to a grasping device (Fig. 1). The curved wire system is made of a shape memory alloy that returns to its original curved state when attached to a lesion, lifting the lesion away from the muscular layer and exposing the submucosa for easier dissection throughout the procedure (Fig. 2). The retraction device–grasper complex is advanced through the working channel of the endoscope and placed on an edge of the target lesion. A second grasper is then used to attach the other side of the wire frame to normal mucosa on the opposite side of the lesion. The second grasper, the anchoring clip, can be placed on the same wall, opposite wall, or side wall from the lesion. The placement of the anchoring clip is chosen based on the lesion and where the endoscopist finds the best traction. The wire frame returns to its curved state, retracting the lesion and exposing the submucosa for dissection. During dissection, the angle of retraction can be changed by placing a second anchoring clip on the wire frame and attaching it to a different area of mucosa at the angle desired. When resection is completed, the entire lesion and device can be removed together by grabbing the grasping device on the normal mucosa or wire frame with a snare or grasping forceps. The second grasping device attached to the normal mucosa is designed to be less traumatic than normal clips on removal because the clip has no teeth.

## Video description

These videos (Video 1, available online at www.giejournal.org) introduce the traction wire technology and demonstrate its use in 3 ESD cases: first, a polyp with high-grade dysplasia in the antrum of the stomach; second, a granular cell tumor in the mid-esophagus; and third, a colon polyp with suspected submucosal involvement. First, the retraction device–clip complex is deployed onto the target lesion. We then demonstrate submucosal dissection with the device in place. Notably, the device provides retraction in the correct direction in all phases of ESD and does not interfere with endoscope movement. The retraction device also allows improved visualization of blood vessels, allowing for easier coagulation of vessels. Because of the simplicity of its design, the device can be deployed anywhere in the GI tract that a clip can be placed, including in retroflexion and in the esophagus. In the first 2 procedures (gastric, esophageal) the anchoring clip is placed on the same side as the lesion, and in the third video (colon), we demonstrate the device when the anchoring clip is placed on the opposite wall of the lesion.

Finally, the video shows a table of the initial 17 ESD procedures that we performed with the traction wire. Deployment and use of the device were successful and beneficial in all cases. Median device deployment time was 2 (1.2-4) minutes. The median procedural time was 58 (41-70) minutes. All procedures achieved en bloc and R0 resection, and there were no perforations.

## Conclusion and implications

Retraction by a curved wire system is simple to use and delivers continuous retraction throughout the ESD procedure. Initial case results have been encouraging.

## Disclosure

*Dr Bhatt is a consultant for Medtronic, Boston Scientific, Steris, and Lumendi and receives royalties from Medtronic. All other authors disclosed no financial relationships.*

## Supplementary data

Video 1 Traction wire presentation and demonstration of technology in 3 endoscopic submucosal dissection cases.
